# A community-based study of demographics, medical and psychiatric conditions, and gender dysphoria/incongruence treatment in transgender/gender diverse individuals

**DOI:** 10.1186/s13293-020-00332-5

**Published:** 2020-10-06

**Authors:** Haleigh A. James, Alice Y. Chang, Reese L. Imhof, Aradhana Sahoo, Monique M. Montenegro, Nicole R. Imhof, Cesar A. Gonzalez, Aida N. Lteif, Todd B. Nippoldt, Caroline J. Davidge-Pitts

**Affiliations:** 1 Division of Endocrinology, Diabetes, Metabolism, and Nutrition, Mayo Clinic, 200 First Street SW, Rochester, MN 55905 USA; 2 Mayo Clinic Alix School of Medicine, Mayo Clinic, 200 First Street SW, Rochester, MN 55905 USA; 3 Department of Psychology and Psychiatry and Department of Family Medicine, Mayo Clinic, 200 First Street SW, Rochester, MN 55905 USA; 4 Division of Pediatric Endocrinology and Metabolism, Mayo Clinic, 200 First Street SW, Rochester, MN 55905 USA

## Abstract

**Background:**

Current understanding about health care in the gender diverse population is limited by the lack of community-based, longitudinal data, especially in the USA. We sought to characterize a community-based cohort of transgender individuals including demographics, gender identities, social characteristics, psychiatric and medical conditions, and medical therapy for gender dysphoria/incongruence.

**Patients and methods:**

We performed a retrospective chart review of gender diverse residents of Olmsted County, Minnesota, who sought gender-specific healthcare from January 1, 1974, through December 31, 2015, using an infrastructure that links medical records of Olmsted County residents from multiple institutions.

**Results:**

The number of patients seeking gender-specific healthcare increased from 1 to 2 per 5-year interval during the 1970s–1990s to 41 from 2011 to 2015 (*n* = 82). Forty-nine (59.8%) were assigned male sex at birth (AMAB), 31 (37.8%) were assigned female (AFAB), and 2 (2.4%) were intersex. Gender identities evolved over time in 16.3% and 16.1% of patients AMAB and AFAB, respectively, and at most recent follow-up, 8.2% and 12.9% of patients AMAB and AFAB, respectively, were non-binary. Depression affected 78%, followed by anxiety (62.2%), personality disorder (22%), and post-traumatic stress disorder (14.6%). 58.5% experienced suicidal ideation, 22% attempted suicide, and 36.6% were victims of abuse. The most prevalent medical conditions and cardiovascular (CV) risk factors included obesity (42.7%), tobacco use (40.2%), fracture [34.1% (86.2% traumatic)], hypertension (25.6%), hyperlipidemia (25.6%), and hypertriglyceridemia (15.9%). 67.3% of patients AMAB used feminizing and 48.4% of patients AFAB used masculinizing hormone therapy. When compared to US CDC National Health Statistics, there was a significantly greater prevalence of depression and anxiety but no difference in the prevalence of obesity, hypertension, hypercholesterolemia, type 2 diabetes, or stroke.

**Conclusion:**

Transgender and gender diverse individuals represent a population who express various gender identities and are seeking gender-specific healthcare at increasing rates. Psychiatric illness is highly prevalent compared to the US population but there is no difference in the prevalence of CV risk factors including obesity, type 2 diabetes, hypertension, and dyslipidemia.

## Introduction

Transgender and gender diverse individuals have incongruence between gender identity and sex recorded at birth. This incongruence can lead to clinically significant distress or the impaired ability to function in social, occupational, or other important areas [1]. Transgender and gender diverse individuals can be marginalized by negative social stigma and prejudices [2], and their health needs have often been ignored. In the last decade, people who identify as transgender and gender diverse have been better recognized as a gender minority, which has spurred much needed research about gender-specific health. Epidemiologic studies have repeatedly shown high rates of adverse health outcomes in this population including psychiatric illness such as mood disorders, anxiety [3], self-injurious behavior, and suicide attempts [4, 5]; HIV [6, 7] and other sexually transmitted infections (STIs); substance abuse [8–10]; and disability [11]. Despite the surge in gender-specific health research in recent years, our knowledge and understanding about this diverse population continues to be limited by the relative lack of community-based, longitudinal data with prior studies from European cohorts or one US-based Western and Southern managed care health care organizations from predominantly more urban centers [12]. There are several reasons for this including poor utilization of healthcare services by transgender and gender diverse individuals because of lack of health insurance or limited coverage for gender-specific health services, as well as avoidance of healthcare facilities due to fear of mistreatment [13], all of which have made it difficult to form research databases. Furthermore, although gender-specific research has previously focused on behavioral and sexual health, substance use and abuse, and social stigma and discrimination, risk for medical conditions remains understudied.

The Rochester Epidemiology Project (REP) is a population-based cohort of Olmsted County residents linking medical records in multiple health care sites throughout Southeast Minnesota and Wisconsin and demonstrating excellent longitudinal follow-up data and characterization of psychiatric and medical conditions with over 90% of residents returning for follow-up visits within 3 years and low attrition rates from the community [14, 15]. In this study, we sought to characterize a community-based cohort of transgender and gender diverse individuals seeking gender-related health care in the Upper Midwest communities of Olmsted County, Minnesota, from 1974 through 2015 including demographics, social characteristics, and the prevalence of psychiatric and medical conditions. We also sought to determine the evolution of gender identities over time, as well as utilization of medical and surgical therapies for gender dysphoria/incongruence.

## Patients and methods

### Patients and setting

This study was approved by the Mayo Clinic and Olmsted Medical Center Institutional Review Boards (IRBs) who review all REP research proposals to ensure that the rights and safety of study participants are protected. Additionally, all REP studies must comply with Minnesota Research Authorization (Minnesota State privacy law—statute 144.335, 1997). This state statute requires that individuals provide permission for their medical records to be used for research studies [16].

We performed a retrospective chart review of transgender and gender diverse residents of Olmsted County, Minnesota who sought gender-specific medical care from January 1, 1974, through December 31, 2015, using the REP [16], an infrastructure that links medical records of Olmsted County residents from multiple institutions which facilitates population-based research. We utilized a standard data extraction form Research Electronic Data Capture (REDCap). Patient charts were reviewed initially by a single reviewer with secondary review by the senior authors if there was a question about a diagnosis. Gender-specific medical care is defined as a medical visit with intention to discuss gender incongruence as primary concern and could be performed by either a medical provider or mental health specialist. By utilizing the REP, we were able to identify and characterize a population-based cohort of transgender and gender diverse individuals presenting for care to any of the participating clinics, hospitals and medical facilities in Minnesota and Wisconsin and who agreed to share their medical records for research. We used billing codes (gender dysphoria, gender identity disorder, psychosexual identity disorder, and transsexualism) to identify possible transgender and gender diverse individuals and only included those patients who were documented to have transgender and gender diverse identities in clinical notes. Patients in this study were included if they had at least one follow-up visit in addition to their initial presentation so that we could determine evolution of gender identities over time, and monitor treatment response with hormone therapy. We comprehensively reviewed these individuals’ medical records from the date of first gender-specific healthcare visit until most recent follow-up and collected the following information.

### Demographics and gender identity

We recorded age at initial visit, race, sex recorded at birth, and gender identity as documented in clinical notes at both initial visit and most recent follow-up. When recording gender identities, we used the following pre-specified categories: male; female; both male and female; neither male or female; gender-queer or fluid; other, indicating different gender identities which did not fit into any of our pre-specified categories; and unclear if we could not determine gender identity from the documentation.

### Psychiatric conditions and events

We recorded the following psychiatric diagnoses documented at any time by healthcare professionals: depression, anxiety, personality disorder, post-traumatic stress disorder, eating disorder, schizophrenia, and autism. We also included the following psychiatric events if they were documented to have occurred in any of the clinical notes: suicidal ideation, suicide attempt, non-suicidal injuring behavior, and psychiatric hospitalization. These predetermined categories were selected based on previous prevalence data in gender diverse individuals [3–5].

### Medical conditions

We recorded the following medical conditions if they affected an individual at any point during their life and were documented as diagnoses in any clinical notes: obesity, hypertension, hypercholesterolemia, hypertriglyceridemia, impaired fasting glucose or prediabetes, type 1 diabetes, type 2 diabetes, pulmonary embolism, deep venous thrombosis, cerebrovascular accident, myocardial infarction, osteoporosis, osteopenia, fracture, cancer, human immunodeficiency virus (HIV), and non-HIV STIs. We also categorized patients as having the following medical conditions if they met the following criteria based on our review of their height, weight, medications, and laboratory results, even if they were not documented to have those specified conditions in clinical notes: obesity defined by body mass index (BMI) ≥ 30 kg/m^2^ in adults or BMI percentile ≥95th percentile for age and sex in patients younger than 18 years; hypertension defined by use of anti-hypertensive medications or if blood pressure was > 140/90 on multiple occasions. Hypercholesterolemia defined by total cholesterol > 239 mg/dL or LDL > 159 mg/dL and/or treatment with lipid-lowering medication such as a statin; hypertriglyceridemia defined by triglycerides > 199 mg/dL and/or treatment with triglyceride-lowering medication such as a fibrate; impaired fasting glucose or prediabetes defined by fasting glucose 100–125 mg/dL, hemoglobin A1c 5.7–6.4%, or glucose of 140–199 mg/dL after a 2-h 75 gram glucose tolerance test; osteoporosis defined by a fragility fracture or T-score ≤ − 2.5 on bone densitometry; and osteopenia defined by a T-score between − 1 and − 2.4. Fractures were categorized as fragility fractures if they affected the hip, vertebra, humerus, or radius after low impact such as a fall from standing height while they were categorized as traumatic if they were clearly caused by a traumatic event such as a motor vehicle accident. We did not formally evaluate the relationship between medical conditions and hormone therapy.

We used data retrieved from the CDC National Health Statistics on the prevalence of medical conditions from 2010 to 2015 [17–19] to determine if the prevalence of medical conditions in transgender/gender diverse individuals was significantly different than the proportions observed in the US population.

#### Health risk behaviors

We recorded history of abuse (sexual, emotional, or physical), previous or current substance use (tobacco smoking, alcohol abuse/dependence, or illicit drug use), and any history of engaging in sex work, as documented in clinical notes.

#### Therapies for gender dysphoria/incongruence

We recorded medical and surgical therapies used. Typical medical therapies for gender dysphoria/incongruence depend on the age of the individual. Adolescents can receive puberty suppression which includes gonadotropin-releasing hormone (GnRH) agonists or progestins. Hormone therapy with sex steroids includes estradiol and testosterone for assigned male at birth (AMAB) and assigned female at birth (AFAB) patients, respectively. Androgen blockade in the USA is typically spironolactone, but GnRH agonists have also been used in individuals who cannot tolerate spironolactone or require a lower dose of estradiol. Feminizing surgical procedures include facial feminization, breast surgery, orchiectomy, and vaginoplasty. Masculinizing surgical procedures include chest surgery, total abdominal hysterectomy and/or bilateral salpingo-ophorectomy, metoidioplasty (release of clitoris to form phallus), and phalloplasty (creation of neophallus). We also determined whether patients received behavioral health evaluations and continuous counseling specifically for gender dysphoria/incongruence.

### Statistical analysis

Statistical analysis was performed using JMP® Pro 14.1.0 (JMP, SAS Institute Inc., Cary, NC, USA). The Fisher’s exact test was used to compare the prevalence of co-morbid conditions between AFAB and AMAB. We compared proportions of medical conditions observed in the transgender/gender diverse cohort to the proportions observed in the US population using the Pearson Chi-Square Goodness of Fit test.

## Results

**Fig. 1:**
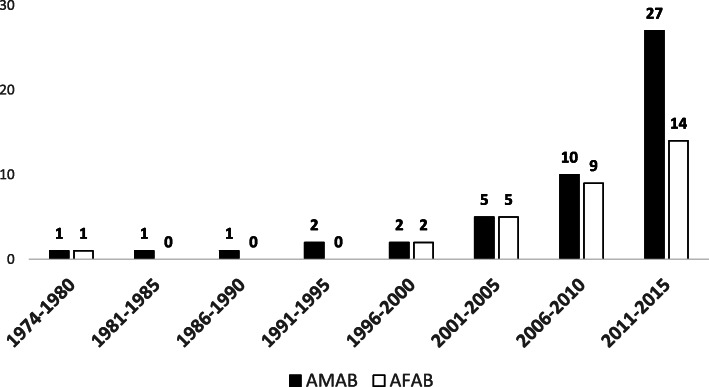
Number of patients seeking gender-specific healthcare in Olmsted County over time, with at least 2 visits

We identified 82 transgender and gender diverse individuals who sought gender-specific medical care between 1974 and 2015 through primary care, psychiatry or endocrinology providers (median follow-up interval 40.5 months, range 1–506). The number of patients increased dramatically from 1 to 2 per 5-year interval during the 1970s–1990s to 41 from 2011 to 2015 (Fig. 1). The largest number of patients (21) presented for care in 2015, correlating with the establishment of the Transgender and Intersex Specialty Care Clinic at Mayo Clinic in Rochester, Olmsted County, Minnesota.

### Demographics and gender identity

The demographics of our cohort are shown in Table 1. Median age at presentation was 24 years (range 5–74). The majority of patients were white. Comparing to data from the REP, this was not significantly different than the Olmsted County population during the study period (91.5% vs 87.8%, *p* = 0.31). 59.8% were assigned male at birth (AMAB), 37.8% were assigned female at birth (AFAB), and 2.4% were intersex.

**Table 1:** Demographic characteristics of cohort

*n* = 82	
Age at initial visit, years, median (range)	24 (5–74)
Age at baseline, years	22 (26.8%) patients < age 18; 56 (68.3%) patients between ages 18 and 50; and 4 (4.9%) patients > age 50
Sex recorded at birth, *n* (%)	49 (59.8) male, 31 (37.8) female, and 2 (2.4) intersex
Race, *n* (%)	75 (91.5%) White; 3 (3.7%) Black; 1 (1.2%) Asian; 1 (1.2%) American Indian; and 2 (2.4%) other/unknown

Gender identity was not always clearly documented or easy to ascertain from clinical notes. Additionally, it was not always binary or stable over time, as shown in Table 2. At the first medical visit, 32 of the 49 (65.3%) patients AMAB identified as female, 6 (12.2%) as gender-queer/fluid, 1 (2%) as male but questioning, 1 (2%) as both male and female, 6 (12.2%) as other, and 3 (6.1%) were unclear. At most recent follow-up, 8 (16.3%) patients’ gender identities had evolved to female, including the patient who identified as male but questioning, the patient who identified as both male and female, 2 of the patients who identified as gender-queer or fluid, and 4 who identified as other at the initial visit. Of the 31 patients AFAB, 29 (93.5%) identified as male, 1 (3.2%) as gender-queer/fluid, and 1 (3.2%) as other at the first medical visit. At most recent follow-up, 5 (16.1%) of these patients had different gender identities documented compared to their initial visits, including 4 who initially identified as male, 2 of whom later identified as female, 1 who later identified as both female and male, and 1 who later identified as other. The patient who initially identified as gender-queer/fluid identified as neither gender at most recent follow-up.

**Table 2:** Gender identity over time (median follow-up interval 40.5 months, range 1–506)

	Assigned male sex at birth, *n* = 49	Assigned female at birth, *n* = 31
Gender identity documented at first medical visit	Female, 32 (65.3%); gender-queer/fluid, 6 (12.2%); male (questioning), 1 (2%); both, 1 (2%); other, 6 (12.2%); and unclear, 3 (6.1%)	Male, 29 (93.5%); gender-queer/fluid, 1 (3.2%); and other, 1 (3.2%)
Gender identity documented at most recent follow-up	Female, 38 (77.6%); gender-queer/fluid, 4 (8.2%); and unclear, 7 (14.3%)	Male, 24 (77.4%); female, 2 (6.5%); neither, 1 (3.2%); both, 1 (3.2%); other, 2 (6.5%); and unclear, 1 (3.2%)

The 2 intersex individuals in our cohort are not included in this table

### Psychiatric conditions and events

As Table 3 shows, psychiatric illness was extremely common in our cohort. The majority of patients were affected by depression and anxiety while almost half of the cohort was diagnosed with personality, post-traumatic stress, bipolar, and eating disorders, schizophrenia or autism. Approximately 40% were hospitalized at some point for psychiatric reasons and approximately 20% had attempted suicide at least once. Suicidal ideation was significantly more prevalent in patients AMAB while non-suicidal injuring behavior was more common in patients AFAB. When compared to age-adjusted United States (US) National Health statistics survey data on depression and anxiety, the prevalence was significantly greater than expected (depression: transgender/gender diverse 77.5% vs US 16.0%, *P* < .01), anxiety (transgender/gender diverse 62.5% vs US 14.4%, *P* ≤ .01).

**Table 3:** Psychiatric/medical conditions and social characteristics of cohort according to sex recorded at birth

	Assigned male at birth (AMAB), *n* = 49	Assigned female at birth (AFAB), *n* = 31	*P* value
Psychiatric conditions			
Depression	38 (77.6%)	24 (77.4%)	1.0
Anxiety	30 (61.2%)	20 (64.5%)	0.82
Personality disorder	13 (26.5%)	4 (12.9%)	0.17
Post-traumatic stress disorder	7 (14.3%)	4 (12.9%)	1.0
Bipolar disorder	7 (14.3%)	3 (9.7%)	0.83
Eating disorder	6 (12.2%)	4 (12.9%)	1.0
Schizophrenia	4 (8.2%)	0	0.15
Autism	3 (6.1%)	1 (3.2%)	1.0
Psychiatric events			
Suicidal ideation	33 (67.3%)	13 (41.9%)	0.04
Suicide attempt	11 (22.4%)	6 (19.4%)	0.79
Non-suicidal injuring behavior	13 (26.5%)	11 (35.5%)	0.46
Psychiatric hospitalization	20 (40.8%)	12 (38.7%)	1.0
Medical conditions			
Obesity	21 (42.9%)	13 (41.9%)	1.0
Fracture	20 (40.8%)	8 (25.8%)	0.23
Hypertension	13 (26.5%)	8 (25.8%)	1.0
Hypercholesterolemia	17 (34.7%)	4 (12.9%)	0.04
Hypertriglyceridemia	11 (22.4%)	2 (6.5%)	0.03
Impaired fasting glucose	8 (16.3%)	1 (3.2%)	0.14
Type 2 diabetes	5 (10.2%)	1 (3.2%)	0.40
Cerebrovascular accident	2 (4.1%)	1 (3.2%)	1.0
Deep vein thrombosis	3 (6.1%)	0	0.28
Pulmonary embolism	1 (2%)	0	1.0
Myocardial infarction	0	1 (3.2%)	0.39
Osteopenia	3 (6.1%)	0	0.28
Osteoporosis	1 (2%)	0	1.0
Cancer	3 (6.1%)	0	0.28
Non-HIV STI	4 (8.2%)	3 (9.7%)	1.0
HIV	0	0	1.0
Health risk behaviors			
History of any abuse	19 (38.8%)	10 (32.3%)	0.64
Emotional	11 (22.4%)	5 (16.1%)	0.56
Physical	9 (18.4%)	4 (12.9%)	0.76
Sexual	9 (18.4%)	8 (25.8%)	0.58
Previous or current substance use			
Tobacco smoking	22 (44.9%)	9 (29%)	0.17
Alcohol abuse/dependence	8 (16.3%)	6 (19.4%)	0.77
Illicit drug use	16 (32.7%)	11 (35.5%)	0.81
Any history of engaging in sex work	2 (4.1%)	0	0.52

Data presented as *n* (%)

Numbers represent the numbers of patients who were documented to be affected by specified conditions or events. They do not represent the actual number of events

*STI* sexually transmitted infection, *HIV* human immunodeficiency virus

### Medical conditions

Obesity was the most common medical condition, affecting 35 (42.7%) of patients in the entire cohort. Six of these patients were diagnosed with obesity before age 18, based on their BMI percentiles, 4 AFAB and 2 AMAB. There was no significant difference in the prevalence of medical conditions between groups except for a higher prevalence of hypercholesterolemia and hypertriglyceridemia in the patients AMAB (Table 3). All of these diagnoses were made in adult patients except for 1 AMAB who was diagnosed with hypercholesterolemia and 2 AFAB who were diagnosed with hypertension before age 18. These diagnoses were made by pediatric specialists and documented in their notes.

Venous thromboembolism (deep venous thrombosis and pulmonary embolism), cerebrovascular accidents, and myocardial infarctions were rare (Table 3). When they occurred, only 3/8 events occurred while on hormone therapy (Table 3). Fractures were common, with 40.8% of patients AMAB and 25.8% of patients AFAB sustaining at least one fracture, although osteopenia and osteoporosis diagnoses were infrequent. Of the 28 patients who sustained fractures, 25 of them sustained traumatic fractures while only one had a fragility fracture and 3 had unknown fracture types. While HIV was not diagnosed in any patients, non-HIV STIs were fairly common, affecting 8.2% of patients AMAB and 9.7% of patients AFAB.

These was no significant difference in age-adjusted US prevalence data for obesity (transgender 42.5% vs US 37.7%, *P* = .38), hypertension (transgender 26.3% vs US 30.2%, *P* = .44), hypercholesterolemia (transgender 26.3% vs US 26.9%, *P* = .90), type 2 diabetes (transgender 7.5% vs US 9.4%, *P* = .56), or stroke (transgender 3.8% vs US 2.5%, *P* = .47). Only the prevalence of prediabetes was significantly lower among transgender individuals (transgender 11.3% vs US 33.9%, *P* < 0.01).

### Health risk behaviors

More than one third of the patients in our cohort had experienced some form of abuse, with emotional abuse being most common in patients AMAB and sexual abuse being most common in those AFAB. Substance use was also highly prevalent with 40.2% of the entire cohort being either former or current smokers, 18.3% abusing alcohol, and 32.9% using illicit drugs. Smoking was more common in patients AMAB compared to those AFAB though the difference was not statistically significant. Marijuana was by far the most commonly used drug with 30.5% of the entire cohort using it, followed by prescription opioids, which were used by 7.3%. Between 1.2 and 3.7% of patients used methamphetamines, cocaine, heroin, ecstasy, hallucinogens, prescription stimulants, and cold medicine each. Two patients endorsed engaging in sex work, both AMAB.

### Therapies for gender dysphoria/incongruence

As Table 4 shows, the majority of patients in our cohort underwent a behavioral health evaluation and received subsequent counseling related to gender dysphoria/incongruence. Among the 49 patients AMAB, 33 (67.3%) used feminizing hormone therapy. Estrogen was most often used, followed by spironolactone, and a minority of patients used progestins, finasteride, or gonadotropin-releasing hormone (GnRH) agonists. 20.4% of patients AMAB underwent feminizing surgical therapy including orchiectomy (14.3%), vaginoplasty (12.2%), breast augmentation (6.1%), and feminizing facial surgery (2%). Among the 31 patients AFAB, 15 (48.4%) used testosterone and 2 also used a progestin. Hormone therapy was typically prescribed by the medical providers documented in the clinical encounters. 32.3% of patients AFAB underwent masculinizing surgical therapy including mastectomy (29%), hysterectomy (19.4%), oophorectomy (12.9%), and phalloplasty (3.2%).

**Table 4:** Medical therapy for gender dysphoria/incongruence

	Assigned male at birth (AMAB), *n* = 49	Assigned female at birth (AFAB), *n* = 31
Behavioral health therapy		
Behavioral health evaluation for gender dysphoria/incongruence	47 (95.9%)	27 (87.1%)
Continuous counseling for gender dysphoria/incongruence	43 (87.8%)	25 (80.6%)
Hormone therapy	33 (67.3%)	15 (48.4%)
Type of hormone therapy	Estrogen: 31 (63.3%) - Age 16-18 years n = 4/31 Spironolactone: 28 (57.1%) - Age 16-18 years n = 3/28 Progestin: 9 (18.4%) - Age 16-18 years n = 1/9 Finasteride: 2 (4.1%) GnRH agonist: 1 (2%) - Age 16-18 years n = 1	Testosterone: 15 (48.4%) - Age 16-18 years n = 1/15 Progestin: 2 (6.5%) GnRH agonist: 1 (3.2%) - Age 16-18 years n = 1
Surgical therapy	10 (20.4%)	10 (32.3%)
Type of surgery	Orchiectomy: 7 (14.3%) Vaginoplasty: 6 (12.2%) Breast augmentation: 3 (6.1%) Facial surgery: 1 (2%)	Mastectomy: 9 (29%) Hysterectomy: 6 (19.4%) Oophorectomy: 4 (12.9%) Phalloplasty: 1 (3.2%)

Data presented as *n* (%)

*GnRH* gonadotropin-releasing hormone

## Discussion

This study provides insight about the transgender and gender diverse community seeking gender-specific healthcare in Olmsted County, Minnesota, dating back to 1974. It shows that transgender and gender diverse individuals represent a population who may seek out gender-specific healthcare at virtually any age. The youngest patient in our cohort presented at age 5 while the oldest presented at age 74. The number of individuals seeking gender-specific healthcare has increased approximately 40-fold between 1974 and 2015 in Olmsted County. This is in the setting of the Olmsted County population increasing less than 2-fold from 88,913 in 1970 to 151,334 in 2015 [20]. We hypothesize that the significant rise in individuals seeking gender-specific healthcare out of proportion to the Olmsted County population increase is a result of growing awareness and acceptance of the transgender and gender diverse community, as well as the establishment of the Transgender and Intersex Specialty Care Clinic at Mayo Clinic in Rochester, Minnesota, in 2015.

Gender identity proved to be complex and sometimes dynamic. Many in our cohort expressed non-binary gender identities, some of which did not fit into our pre-specified categories. This supports the fact that gender identity can fall anywhere on a spectrum and can even encompass multiple genders or none. It is also interesting to note that gender identity evolved over time in approximately 16% of individuals. In most cases, such as the 8 patients AMAB who initially endorsed various non-female gender identities but later expressed female gender identities, this seemed to represent an evolution of gender identity realization rather than desistence. Although 2 of the patients AFAB who initially identified as male later identified as female, we cannot say for sure that this necessarily represented gender desistence. Given that documentation regarding gender identity in these cases was limited, it is possible that these individuals endorsed cisgender identities at most recent follow-up because of social pressures or other contextual factors not clearly documented in medical notes. Our data, which illustrates the complexity and potentially dynamic nature of gender identity realization, emphasizes the importance of supportive counseling for individuals who are questioning their gender identities.

Similar to previous studies [3, 4, 10, 11], behavioral health issues including self-harm and psychiatric hospitalization, history of abuse, and substance use were highly prevalent in our cohort, reinforcing the crucial role of behavioral health providers in multidisciplinary care teams who treat these patients. In contrast to previous studies showing high rates of HIV in transgender persons [6, 7], HIV was not diagnosed in any of our patients, which is likely a result of its low prevalence in Olmsted County [21], but other STIs were not uncommon.

Our review of medical conditions shows that obesity was very common, affecting greater than 40% of the cohort. The reasons for this are unclear, but we suspect they are multifactorial. While hormone therapy may affect fat mass and BMI [1], this cannot be the only contributor in our cohort because many patients did not receive hormone therapy, and of those who did, obesity often predated initiation of hormone therapy. A study evaluating dietary and exercise patterns in transgender individuals showed several obesogenic lifestyle habits including high caloric intake relative to physical activity, diets rich in saturated fat, and skipping breakfast [22]. Disordered body image may also contribute since overweight and obese transgender youth have been found to be more likely to view themselves as normal weight or underweight compared to cisgender youth [23]. We hypothesize that the high prevalence of psychiatric illness, especially depression, may be a potential contributor because it can lead to excessive eating as a coping mechanism. Additionally, several psychotropic medications such as antipsychotics can cause weight gain. Eating disorders affected more than 12% of our cohort. While some eating disorders such as anorexia nervosa may cause low body weight, others such as binge eating disorder may have contributed to obesity in a subset of our patient population. A high prevalence of social anxiety among transgender/gender diverse individuals might also contribute to a decrease in exercise and physical activity in public settings such as gyms or parks as misgendering and gender discrimination is a significant trigger for psychological distress [24].

Several other cardiometabolic risk factors including smoking, hypertension, hyperlipidemia, hypertriglyceridemia, and impaired fasting glucose affected a significant proportion of transgender individuals. This suggests that the transgender and gender diverse population may be at elevated risk for more serious cardiometabolic conditions such as atherosclerotic cardiovascular (CV) disease and type 2 diabetes. Although our data shows much lower rates of these latter conditions, we must acknowledge that the median age of our patients at initial presentation was relatively young and some individuals who presented for gender-specific healthcare in recent years have not had prolonged follow-up, so it is possible that they may go onto develop these conditions. Furthermore, we did not compare our cohort with a matched cisgender cohort, so we cannot say if the rates of medical conditions in our transgender cohort are higher or lower than those of the cisgender population. In comparing the prevalence of medical conditions to available US National Health statistics survey data, there was no significant difference in proportions observed in the transgender cohort to the US population except a lower prevalence of prediabetes. However, the lack of routine screening might have led to underdiagnosis in our cohort and highlights the need to improve access to health care for transgender individuals. Regardless of these limitations, while our reported rates of obesity and other CV risk factors in this predominantly adult cohort were no different than the US population, they are important health concerns for all US adults and highlight the importance of medical providers screening for and treating these conditions appropriately, especially since some forms of hormone therapy may exacerbate them. A high prevalence of fractures has not been previously reported, especially in patients AMAB. Given that the majority of fractures were traumatic rather than fragility fractures, however, we were not able to determine if they were due to high risk behavior and/or physical altercations.

Our assessment of medical therapy for gender dysphoria/incongruence shows that the majority of transgender and gender diverse patients who sought gender-specific healthcare between 1974 and 2015 received behavioral health evaluations and continuous counseling. Feminizing hormone therapy was used by 2/3 of the patients AMAB and approximately half of the patients AFAB, while a minority of individuals received surgical therapy for gender dysphoria/incongruence. The greater proportion of AMAB receiving hormone therapy might explain the significantly greater prevalence of hyperlipidemia/hypertriglyceridemia seen in AMAB as both feminizing and masculining therapy have been associated with dyslipidemia [25]. These numbers may represent the fact that not all transgender and gender diverse individuals desire feminizing or masculinizing hormone therapy or surgery, but they are more likely a result of inadequate access to these therapies. Education and comfort prescribing feminizing or masculinizing hormone therapy among medical providers has been limited [26], and few surgeons have been trained in gender-affirming surgery. Insurance coverage has also likely been a barrier for many patients. As the principles of feminizing or masculinizing hormone therapy are being incorporated into medical education, increasing numbers of providers become comfortable prescribing it, and more surgeons are trained to perform gender-affirming surgeries, future studies can determine if increased access to gender-specific healthcare changes the prevalence of mental health disorders and medical conditions. Strengths of this study include the ability to gather data over many decades with individual data available for a median of 40.5 months of follow-up and a wider age range, which improves our ability to look at diseases affecting an older population such as cardiovascular disease and stroke. A large volume of prior data about health risks and comorbidities of individuals with gender dysphoria/incongruence come from European cohorts [27] which may not be representative of the US population. We present a cohort that is US and Midwest specific, which may provide additional data to other US cohorts that are from the West or South and in a single managed care network (Kaiser Permanente) [12]. In comparison to current US data from the Kaiser study on transgender and gender diverse individuals, our cohort has predominance of white individuals (91.5% vs 55%) and a higher prevalence of obesity (42.7% vs 26%). Our data also reveals a higher prevalence of mental health disorders such as anxiety (in AMAB 61.2% vs 38%), depression (in AMAB 77.6% vs 49%), self-injury (in AMAB 26.5% vs 2.2%), and suicidal ideation (in AMAB 67.3% vs 5%) compared to the Kaiser cohort. We were able to capture all patients diagnosed with gender dysphoria/incongruence, not just those enrolled in a specific hormone and surgery protocol such as those in Europe. For example, 86.7% of the Dutch cohort has undergone “sex reassignment surgery” which includes orchiectomy [28] compared to 12.2% of our cohort and 5.2% of other US cohorts. Therefore, our sample might be considered more relevant to what is seen in community practices.

Our study has some limitations. First, because the focus of our study was on individuals seeking gender-specific healthcare, it does not include all transgender/gender diverse individuals in the community and might have been biased to more severely symptomatic individuals. This is also demonstrated by the almost 40-fold increase in individuals seeking gender-specific health care over the course of the study period. Our study focused on a single geographically defined US population, and the observed associations may differ in other populations in the US and worldwide. However, the demographic and socioeconomic characteristics of the REP population have been shown to be representative to those of the upper Midwest and of a large segment of the entire US population [14] who have not previously been represented in US studies of transgender and gender diverse individuals. Future studies in other gender diverse populations in the US and worldwide will allow for useful comparisons.

We also report that our cohort experienced increased prevalence of mental health concerns. Although this is consistent with previous reports in transgender and gender diverse individuals, diagnosed depression and anxiety could have been related to clinical distress related to gender dysphoria/incongruence, so this study might have been affected by selection bias of individuals with more severe symptoms and greater mental health concerns who presented for treatment. Because of the variability in practice and lack of insurance coverage for hormone therapy in the past, we were not able to provide meaningful information on the doses of hormone or types of hormone therapy. It is also possible that individuals found alternative sources for hormone therapy that they did not report. In addition, due to the lack of consistent collection of biochemical testing, this was not formally analyzed in this cohort. If data were present, we did use metabolic parameters or vital signs for definitions of metabolic conditions. Although this might have led to reporting of a lower prevalence of medical conditions, it would not affect the conclusions regarding the higher prevalence of psychiatric conditions compared to other populations.

## Conclusion

In this community-based study, we show that transgender and gender diverse individuals are seeking gender-specific healthcare at increasing rates in recent years. They represent a diverse patient population who express a variety of gender identities, which may evolve over time. Psychiatric illness is highly prevalent compared to the US population but there is no difference in the prevalence of CV risk factors including obesity, type 2 diabetes, hypertension, dyslipidemia. Future studies are needed to examine the role of improved access to therapies for gender-specific healthcare in decreasing the prevalence psychiatric illness and in the development of CV risk factors

### Perspectives and significance

This is the first US Midwest cohort of transgender and gender diverse individuals who presented for gender-specific healthcare since 1974. There was a significantly greater prevalence of depression and anxiety and no significant difference in cardiovascular risk factors or outcomes compared to the US population though not all participants received hormone therapy or gender-affirming surgical procedures. This study highlights the importance of a multidisciplinary team for optimization of care. Future studies will evaluate how improved access to gender-specific healthcare will affect the prevalence of mental health and medical conditions.

## Data Availability

The datasets generated and analyzed during the current study are not publicly available due to concerns of patient confidentiality but are available from the corresponding author on reasonable request.

## Abbreviations

AFAB: Assigned female at birth
AMAB: Assigned male at birth
BMI: Body mass index
CV: Cardiovascular
HIV: Human immunodeficiency virus
REP: Rochester Epidemiology Project
STI: Sexually transmitted infection
